# Circulating microRNA Panels for Detection of Liver Cancers and Liver-Metastasizing Primary Cancers

**DOI:** 10.3390/ijms242015451

**Published:** 2023-10-22

**Authors:** Branislava Ranković, Nina Hauptman

**Affiliations:** Institute of Pathology, Faculty of Medicine, University of Ljubljana, Korytkova 2, SI-1000 Ljubljana, Slovenia; branislava.rankovic@mf.uni-lj.si

**Keywords:** malignant liver tumors, primary malignant liver tumors, liver metastases, serum, plasma, circulating microRNA, tissue of origin determination

## Abstract

Malignant liver tumors, including primary malignant liver tumors and liver metastases, are among the most frequent malignancies worldwide. The disease carries a poor prognosis and poor overall survival, particularly in cases involving liver metastases. Consequently, the early detection and precise differentiation of malignant liver tumors are of paramount importance for making informed decisions regarding patient treatment. Significant research efforts are currently directed towards the development of diagnostic tools for different types of cancer using minimally invasive techniques. A prominent area of focus within this research is the evaluation of circulating microRNA, for which dysregulated expression is well documented in different cancers. Combining microRNAs in panels using serum or plasma samples derived from blood holds great promise for better sensitivity and specificity for detection of certain types of cancer.

## 1. Introduction

Malignant liver tumors are among the most common malignancies worldwide. They include both primary liver malignancies and liver metastases and are often characterized by poor prognosis and low overall survival rate. Hepatocellular carcinoma (HCC) is the predominant primary liver malignancy, followed by intrahepatic cholangiocarcinoma (CCA) [1,2,3,4]. The incidence of liver metastases exceeds that of primary liver tumors [5]. Liver metastases can arise from a variety of malignancies, including carcinomas, melanomas, lymphomas, sarcomas, and germ cell tumors [5,6]. Among these, carcinomas are the most common (92%), with adenocarcinoma being the most common subtype (75%) [6]. The main sources of liver metastases are colorectal carcinomas, followed by pancreatic, breast, lung, and gastric carcinomas [4,6].

Differentiation between HCC and CCA as well as liver metastasis is usually straightforward. However, occasionally pathologists are challenged by difficult cases, such as small biopsies, different histological subtypes or inconclusive immunophenotype [5]. The interplay of factors such as the etiology of liver disease, the rate of cancer progression, and molecular diversity both between metastatic specimens and within the same tumor mass complicates the differentiation of malignant liver tumors. In some cases, the primary tumor site or tissue of origin (TOO) of a metastatic tumor remains unclear and is therefore classified as a carcinoma of unknown primary (CUP) [6,7]. It should be noted that most CUPs are detected in the liver [7]. The data show that up to 24% to 50% of CUP patients have liver metastases and that these patients are associated with an increased mortality rate [7,8,9]. In addition, patients with liver metastases often have a worse overall survival prognosis compared to patients with metastases to other anatomic sites [3].

Because prognosis and treatment are often based on the type of primary cancer, the differentiation between primary liver malignancies and liver metastases and the determination of TOO in CUP in the liver are of vital importance [1,3,6,10].

## 2. MicroRNA in Cancer

MicroRNAs (miRNAs) represent a class of small non-coding RNAs, typically between 20 and 22 nucleotides in length. These intriguing molecules are derived from various genomic sources, including intergenic regions and exon sequences within non-coding transcription units. It is noteworthy that a substantial proportion, up to 60%, of known miRNAs arise from intronic sequences nestled within either protein-coding genes or non-coding transcription units. MiRNAs can be found encoded as individual genes or clustered together in genomic regions. In some instances, miRNA clusters are co-regulated and co-transcribed, hinting at intricate regulatory mechanisms [11]. In the human genome, miRNAs are estimated to constitute more than 3% of the entire set of genes. Functionally, miRNAs exhibit remarkable diversity, impacting various facets of gene regulation. However, their principal mode of action in mammals primarily involves the inhibition of mRNA translation through base-pairing interactions with the 3’-UTR (untranslated region) of target mRNAs. In the complex milieu of animal cells, individual miRNAs can exert their influence over numerous mRNA targets, sometimes numbering as many as 200 predicted targets per miRNA. Additionally, a single mRNA can be subject to regulation by multiple miRNAs [12].

In the context of cancer research, miRNAs have emerged as pivotal gene-specific regulators, bearing similarities in their activities to a multitude of protein transcription factors known to be crucial players in the transformation of normal cells into malignant ones. MiRNAs wield their influence over various stages of gene expression, including transcription, mRNA stability, and mRNA translation. Notably, cancer cells exhibit genetic and epigenetic alterations compared to their non-malignant counterparts, and miRNAs are increasingly recognized as central players in mediating these distinctions. Genome-wide profiling endeavors have unveiled distinct miRNA signatures unique to specific cancer types, underscoring the diagnostic potential of these molecules. Combining miRNA markers with other biomarkers holds promise for enhancing cancer risk assessment, detection, and prognosis. Moreover, specific genetic polymorphisms have been linked to the susceptibility of developing various types of cancer. Hence, there is a growing imperative to integrate genomic mutations with miRNA markers to formulate comprehensive marker panels that offer more accurate risk assessment and early diagnosis in the realm of cancer research and clinical practice [13,14,15].

## 3. Circulating Tumor miRNA

A significant breakthrough has been the identification of miRNAs as potential biomarkers in serum or plasma, offering a minimally invasive approach to cancer screening. Therefore, understanding the characteristics of secretory miRNAs and their utility in cancer detection is of paramount importance [16,17].

**Figure 1:**
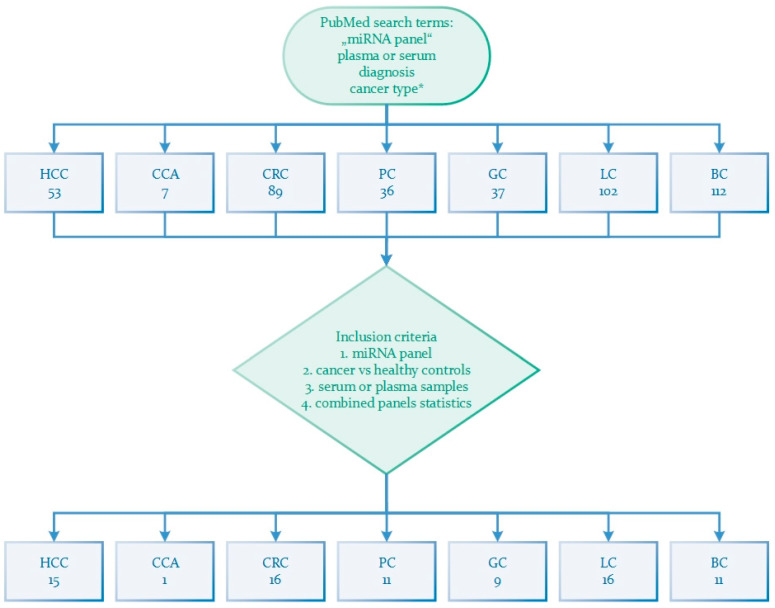
Schematic presentation of workflow. The literature search was performed in PubMed with terms “miRNA panel”, plasma or serum, diagnosis and term for each cancer included in this review. Seven separate searches resulted in different numbers of studies from which we selected those that met the inclusion criteria. HCC: hepatocellular carcinoma; CCA: cholangiocarcinoma; CRC: colorectal cancer; PC: pancreatic cancer; GC: gastric cancer; LC: lung cancer; BC: breast cancer; *: HCC, CCA, CRC, PC, GC, LC, or BC.

In recent years, miRNAs have gained significant attention among researchers exploring their potential as biomarkers for cancer diagnosis and prognosis. To systematically prepare this review paper, we conducted a comprehensive search of the literature on PubMed. We combined the terms “miRNA panel” with “plasma” or “serum” and “diagnosis” for each type of cancer selected in this review; these are the primary liver cancers HCC and CCA and liver-metastasizing cancers colorectal cancer (CRC), pancreatic cancer (PC), gastric cancer (GC), lung cancer (LC), and breast cancer (BC) (Figure 1). In total, seven separate searches were performed.

Our search yielded the following results: HCC 53, CCA 7, CRC 89, PC 36, GC 37, LC 102, and BC 112. We meticulously reviewed these studies and selected those that employed a panel of miRNAs for the detection of each cancer type, distinguishing them from healthy controls. We further selected original articles that tested their selected miRNA panels on human serum or plasma samples and evaluated a combined panels of selected miRNA markers. Furthermore, by combining different miRNAs from each cancer panel, we explore the potential to create a more specific blood-based panel capable of detecting multiple cancers simultaneously, offering a promising avenue for comprehensive cancer screening.

## 4. Circular miRNA in Primary Liver Cancers

### 4.1. Hepatocellular Carcinoma

We categorized miRNA panels into three groups. The first group consisted of eight panels exclusively featuring miRNAs. In the second group, there were three panels where miRNAs were combined with lncRNA and mRNA. The third group involved four panels where miRNAs were combined with α-fetoprotein (AFP).

It is worth noting that several miRNAs were consistently utilized across multiple miRNA panels. These shared miRNAs are miR-126, miR-21, miR-122, miR-125b, miR-375, miR-206, miR-192, miR-223, miR-26a, and miR-27a. Additionally, AFP was a recurring component in different panels (Table 1).

#### 4.1.1. miRNA-Only Panels

In our review of miRNA-only panels for HCC detection, all the panels exhibited high diagnostic accuracy, with area under receiver operating characteristic curve (AUC) values ranging from 0.887 to 1.00 (Table 1). These panels consisted of two to eight miRNAs per panel [18,19,20,21,22,23,24,25]. The development of these miRNA panels followed diverse methodologies. Some researchers utilized microarrays [18] or gene expression arrays [22] for the initial screening process, while others leveraged datasets from the Gene Expression Omnibus (GEO) or The Cancer Genome Atlas (TCGA) [23] or employed sequencing techniques [19]. In the process of panel development, some studies took a more straightforward approach [20,24,25], while others adopted a phased strategy. One study employed a two-phase approach [22], and others extended it to a three-phase approach [18,19,23].

Although some panels exhibited high AUC, like the studies of Ali et al. [24] and Jiang et al. [25], their sample cohorts were quite small, 34 and 27 HCC cases, respectively. To obtain more objective results, these two panels should undergo additional testing. More reliable results are those from studies that used multi-phase testing on larger cohorts, such as the studies performed by Zhou et al. [18], Tan et al. [19] and Zhu et al. [21].

The panel with the most included samples was one from the study of Zhou et al. that used microarrays to screen 723 microRNAs in 137 plasma samples for diagnosing HCC. The panel was tested on a training cohort and then validated using an independent cohort, providing a high diagnostic accuracy for HCC [18].

#### 4.1.2. miRNA Panels Combined with lncRNA and mRNA

Compared to miRNA-only panels, the panels where miRNAs were combined to lncRNA and mRNA had sensitivities from 79.5% to 100% and specificities from 76.7% to 100% (Table 1) [26,27,28]. However, it is worth noting that the statistical data, while promising, were derived from cohorts with relatively smaller sample sizes compared to the miRNA-only panels, with sample numbers ranging from 49 to 78 HCC cases. These studies also followed a straightforward approach without validation cohorts; therefore, they should be tested on independent cohort to confirm their statistical value.

#### 4.1.3. miRNA Panels Combined with AFP

AFP is one of the most widely used biomarkers since it was first introduced in the 1960s; nevertheless, its sensitivity value to diagnose HCC is around 60% and the specificity is still inadequate [33]. In up to one-third of HCC cases, serum levels of AFP remain normal; furthermore, elevation in AFP can also occur in some benign liver diseases as well as other tumors (germinal cell tumor) [34].

AFP was included to improve the statistical value of the panels, which is confirmed by the high value of the AUCs, ranging from 0.936 to 1 (Table 1) [29,30,31,32]. Although none of the panels combined with AFP had a multi-phase approach, the study performed by Zekri et al. had a big cohort of 192 HCC cases, and their panels also exhibited the best statistic potential [29].

### 4.2. Cholangiocarcinoma

For CCA, we could only include one panel, as the other search results did not meet our inclusion criteria, which required a combined statistical score (Table 2).

Wada et al. identified a seven-miRNA panel from publicly available datasets. This panel underwent testing on 241 tissue samples from two clinical cohorts, comprising a training set (*n* = 177), a validation set (*n* = 64), and matched plasma samples (*n* = 68). The panel successfully discriminated CCA from healthy individuals with an AUC of 0.781 [35]. It is worth noting that only three out of seven miRNAs in this panel are unique to CCA, namely miR-219a, miR-338, and miR-421. The other four miRNAs are also found in some of the panels used for discriminating HCC from healthy individuals.

## 5. Circular miRNA in Liver-Metastasizing Primary Cancers

The liver is a common site for metastasis, in part due to its unique and diverse cellular and architectural composition that renders the liver hospitable to tumor cells. According to epidemiology studies, far more common secondary tumor deposits originate from colon and lung cancers. However, pancreatic, gastric, breast and prostate cancers are also known to spread to the liver. Since the presence of liver metastases is associated with worse survival, accurate and prompt diagnosis is crucial [36,37,38]. Not rarely does a pathologist face difficulties in differentiating metastases of unknown origin from primary liver tumors.

### 5.1. Colorectal Cancer

The miRNA panels detecting CRC were divided into two groups. The first group consisted of 13 panels exclusively featuring miRNAs. The second group had three panels, which included miRNAs, lncRNA, and mRNA. In all panels detecting CRC, we found some miRNAs included in more panels; these miRNAs are miR-21, miR-27a, miR-143, and miR-145 (Table 3).

#### 5.1.1. miRNA-Only Panels

Panels demonstrating diagnostic potential for distinguishing between CRC and healthy controls exhibited AUC values ranging from 0.745 to 0.906 (Table 3). In the discovery phase, various methodologies were employed, including bioinformatics utilizing data from GEO and TCGA [42,43,51], gene expression arrays [40,41,47], and sequencing [39]. The studies used two-phase [47], three-phase [40,41,43,45,48,49], and four-phase testing [39,40,42,44,46].

The study by Li et al. stood out with the highest AUC, employing a four-miRNA panel selected using GEO and TCGA data. This panel was validated on both tissue and plasma samples, showing great promise. However, it is important to note that their cohort was relatively modest, consisting of 50 CRC cases. Thus, it is advisable that the panel undergoes independent cohort testing to validate the observed statistics [51].

In contrast, the study by Vychytilova-Faltejskova et al. featured the largest cohort, making it a highly reliable study based on the number of samples. It was conducted in three phases and utilized sequencing for testing. The panel was assessed in a total of 427 CRC patients and 276 healthy donors. The discovery phase, conducted through Illumina small RNA sequencing, identified fifty-four significantly dysregulated microRNAs in the sera of CRC patients compared to healthy individuals (*p*-value < 0.01). This study established a diagnostic four-microRNA signature with an AUC of 0.877, effectively distinguishing early-stage CRC patients from healthy individuals [39].

#### 5.1.2. miRNA Panels Combined with lncRNA

In our exploration, we uncovered three distinct panels that incorporated both miRNA and other RNAs. Among these panels, one particularly stood out, demonstrating an impressive AUC of 0.954. This panel featured a combination of four miRNAs and two lncRNAs, tested on a substantial cohort of 597 CRC patients and 585 healthy controls (HC). These RNAs exhibited notable upregulation in the plasma of CRC patients compared to healthy individuals. This specific panel exhibited exceptional performance with an AUC of 0.996 in the training set and 0.954 in the validation set [52]. However, it is important to note that two additional panels were conducted that integrated miRNA, lncRNA, and mRNA with high sensitivities, albeit they were tested on considerably smaller cohorts [53,54] when compared to the comprehensive panel designed by Li et al. [52].

### 5.2. Pancreatic Cancer

The miRNA panels designed for PC detection can be categorized into three groups: miRNA-only panels, panels combining miRNAs with the carbohydrate antigen 19-9 (CA19-9) tumor marker, and panels combining miRNAs with proteins (Table 4).

Interestingly, several miRNAs consistently appear in multiple panels. These shared miRNAs include miR-16, miR-24, miR-34a, miR-122, miR-130a, miR-145, miR-223, and miR-885. The tumor marker CA19-9 is also included in more panels.

#### 5.2.1. miRNA-Only Panels

A total of six panels exclusively comprised miRNAs for PC detection. The AUCs observed in these panels were between 0.862 and 0.96 (Table 4). The approaches used in the discovery phase of these panels consisted of machine learning methods [57], bioinformatics [60], gene expression arrays [55,56,58,59] and sequencing [57]. The panels were tested in two phases [57], three phases [55,59,60], or four-phases [56,58].

The panel with the best statistic for discriminating between PC and healthy individuals was proposed by Franklin et al. with an AUC of 0.96. This study also had the most miRNAs in the panel—15 miRNAs. The study was designed as a multi-phase study, although it had somewhat smaller sample size and should be further independently tested on larger patient cohorts [59]. The panel with the largest cohort of samples used to discriminate among PC and healthy controls included 409 PC cases and 312 healthy individuals. The panel was composed of 10 miRNAs, and the samples were divided into discovery, training, and validation cohorts. The training cohort exhibited an AUC of 0.93 (95% CI: 0.90–0.96); these results were comparably robust in the validation cohort, with an AUC of 0.93 (95% CI: 0.89–0.97) [55].

#### 5.2.2. miRNA Panels Combined with CA19-9

Although it is one of the most widely used tumor markers, CA19-9 is not exclusive to PC, since its levels may be significantly increased in cases of benign biliary conditions, especially those with obstructive jaundice, as well as some other malignancies such as hepatocellular, gastric, colonic, esophageal, and other non-gastrointestinal cancers, and its interpretation should correlate with other markers [66].

Nevertheless, incorporating CA19-9 into miRNA panels has shown promising results in enhancing the detection of PC. Four notable studies have explored this combined approach. Three of the studies used gene expression arrays in the discovery phase of the study [61,63,64]. Although not having the highest AUC, the most reliable is the panel proposed by Johansen et al., who employed a comprehensive three-phase discovery process. Initially, they utilized the TaqManVR Human MicroRNA assay to identify 34 differentially expressed miRNAs between PC patients and healthy controls. These miRNAs were subsequently tested in a training cohort, leading to the construction of a diagnostic panel comprising 12 miRNAs. Among these, Index III, when combined with CA19-9, exhibited remarkable diagnostic performance with an AUC of 0.94 (0.90–0.97), sensitivity of 85%, specificity of 98%, and overall accuracy of 89% for distinguishing PC patients from healthy individuals [61].

#### 5.2.3. miRNA Panels Combined with Proteins

We included one more panel for detection of PC where four miRNAs were combined with four proteins. The selection process was performed on exosomes of PC cell lines and evaluated on PC exosomes of patients. This panel shows great promise, with a sensitivity of 100% for distinguishing PC from healthy controls [65].

### 5.3. Gastric Cancer

The miRNA panels designed for detection of GC compared to healthy controls can be divided into two groups: those composed solely of miRNAs and those combining miRNAs with the lncRNA (Table 5).

Some miRNAs are included in more panels; these are miR-21, miR-92a, miR-93 and miR-106a.

#### 5.3.1. miRNA-Only Panels

We included eight miRNA-only panels for distinguishing GC from healthy individuals (Table 5). Interestingly, the discovery phase included only two methods; one was gene expression arrays [67,68,70,73] and the other microarrays [74]. The panels were composed of 2 to 12 miRNAs, with a statistical importance of AUC from 0.702 to 0.92 (Table 5). The statistical rigor is better when multi-phase studies are adopted, which was performed in cases with two-phase studies [71,72,73,74], three-phase studies [67,68,69], and four-phase studies [70].

The study with the highest AUC, the largest cohort and the most extensive study we uncovered for detection of GC using miRNA panels was a comprehensive three-phase, multicenter investigation involving a total of 5248 subjects from Singapore and Korea. The biomarker discovery and verification phases were accomplished through comprehensive serum miRNA profiling and multivariate analysis of 578 miRNA candidates in retrospective cohorts of 682 subjects. Subsequently, a clinical assay was developed and rigorously validated in a prospective cohort of 4566 subjects. The culmination of this research effort resulted in the creation of a clinical assay for the detection of GC based on a robust 12-miRNA biomarker panel. This panel demonstrated exceptional performance with an AUC of 0.93 (95% CI: 0.90–0.95) in the discovery cohort and an AUC of 0.92 (95% CI: 0.88–0.96) in the verification cohort. In the prospective study, the assay exhibited an overall sensitivity of 87.0% (95% CI: 79.4–92.5%) at a specificity level of 68.4% (95% CI: 67.0–69.8%), ultimately yielding an AUC of 0.848 (95% CI: 0.81–0.88) [67]. This study has already completed the clinical trial phase and is now available as a test named The GASTROClear. The test is labeled as an in vitro diagnostic medical device (IVD) for the detection of gastric-neoplasia-associated miRNA biomarkers in human serum and has been shown to detect 87% of all GC, including up to 89% of early-stage GC [76].

#### 5.3.2. miRNA Panels Combined with lncRNA

In miRNA panels for GC detection, there was only one panel with a combination of miRNAs and lncRNAs, comprising miR-675-5p, H19, and MEG3. Although Ghaedi et al. tested several miRNAs, this was the final selected panel that could discriminate among GC and healthy subjects with an AUC of 0.927; the sample size used for testing was quite small, therefore further testing should be performed to validate the statistical performance of the test [75].

### 5.4. Lung Cancer

The miRNA panels for the detection of LC were divided into panels with only miRNAs and panels with combination of miRNAs and lncRNAs. Several miRNAs were included in many panels, such as miR-205, miR-215, miR-200b, miR-375, miR-486, and miR-1299 (Table 6).

#### 5.4.1. miRNA-Only Panels

We included 14 studies with miRNA-only panels for detection of LC from healthy individuals (Table 6). Some studies described discovery phases with methods of gene expression array [78,80,88], sequencing [79,86,87], or bioinformatics [81,83,85]. The studies were two-phased [80,82,83,86,87,88,89,90] and three-phased [77,78,79,81,85].

For the detection of LC, the panels comprised from two to six miRNAs per panel, with the statistical significance of AUC from 0.8013 to 1 (Table 6). Although the AUC was 1, we have to acknowledge that the sample size was quite small, with only 30 NSCLC and 20 healthy control samples [89]. In LC, we have three panels with cohort sizes over 400 cases. These studies exhibit AUC from 0.873 to 0.973 [77,78,79].

The biggest cohort study included 1132 participants and was divided into a training cohort (n = 565) and independent validation cohort (n = 461). For the screening phase, a microarray was used that helped identify six microRNAs (miR-17, miR-190b, miR-19a, miR-19b, miR-26b, and miR-375), providing high diagnostic accuracy in discriminating LC patients from healthy individuals with AUCs of 0.873 and 0.868 for the training and validation cohort, respectively [78].

The second biggest cohort included 744 NSCLC cases and 944 matched controls. A miRNA panel for NSCLC detection was developed with validation on three cohorts. Ying et al. discovered 35 candidate miRNAs, of which 22 were verified, and subsequently developed a 5-miRNA panel that detected NSCLC with AUCs between 0.936 and 0.984 in the discovery and verification cohorts. The panel was validated in three independent cohorts with AUCs of 0.973, 0.916, and 0.917. The sensitivity of the miRNA panel was 81.3% [77].

Finally, Yao et al. attempted to construct a miRNA panel for lung adenocarcinoma (LUAD) detection. The starting material was plasma-derived extracellular vesicles (EVs). They observed upregulation of miR-451a, miR-194-5p, and miR-486-5p in EVs from LUAD patients, compared to healthy controls. The AUC of the combined panel was 0.9650 [79].

#### 5.4.2. miRNA Panels Combined with lncRNA

In detection of LC in blood samples, there were two studies that besides miRNA included lncRNA [91,92]. The panel with better statistical value had an AUC of 0.861; although its cohort was larger, it was still modest compared to studies with bigger cohorts described in miRNA-only panels. Therefore, further validation would be needed to confirm the statistical value of the panel [91].

### 5.5. Breast Cancer

We found 12 miRNA panels for detection of BC patients compared to normal, of which only one was composed of miRNAs and lncRNA and the others were composed of only miRNAs. We also identified miRNAs included in more than one panel, which are miR-9, miR-19b, miR-20b, miR-92a, miR-106a, and miR-133a (Table 7).

#### 5.5.1. miRNA-Only Panels

We included 11 studies with miRNA-only panels for the detection of BC from healthy individuals (Table 6). The methods used in the discovery phase were gene expression array [94,95], sequencing [102], bioinformatics [98,99], or microarrays [97,101]. Some studies used two-phase [95,102], three-phase [93,96,97,98], or four-phase [94] approaches. The studies selected for this review had panels for BC detection composed of two to eight miRNAs with statistical values of AUC between 0.8387 and 0.978 (Table 7).

Interestingly, the best statistical value belonging to the panel with the second-largest cohort. Using an Exiqon panel, Li et al. selected candidate miRNAs in a screening phase, followed by analysis in training, testing, and external validation phases. They identified five plasma miRNAs with significantly different expression levels between BC patients and healthy individuals. This panel achieved AUCs of 0.683, 0.966, and 0.978 for the training, testing, and external validation sets, respectively [94].

Among the studies of miRNA panels for BC detection, several had large cohorts. The larges was divided into cohorts of discovery phase (n = 289) and two validation phases (n = 374 and n = 379). The researchers identified and validated 30 miRNAs with dysregulated expression in BC. An optimized eight-miRNA panel consistently performed well across all cohorts, achieving an AUC of 0.915, accuracy of 82.3%, sensitivity of 72.2%, and specificity of 91.5% [93].

#### 5.5.2. miRNA Panels Combined with lncRNA

Only one panel in BC was a combination of miRNAs and lncRNAs. The study was conducted in two phases, consisting of training and validation sets. The selected panel consisted of three miRNAs and one lncRNA. The AUCs were 0.960 and 0.968 for the training and validation sets, respectively [103].

## 6. miRNA Specificity in Cancers

**Figure 2:**
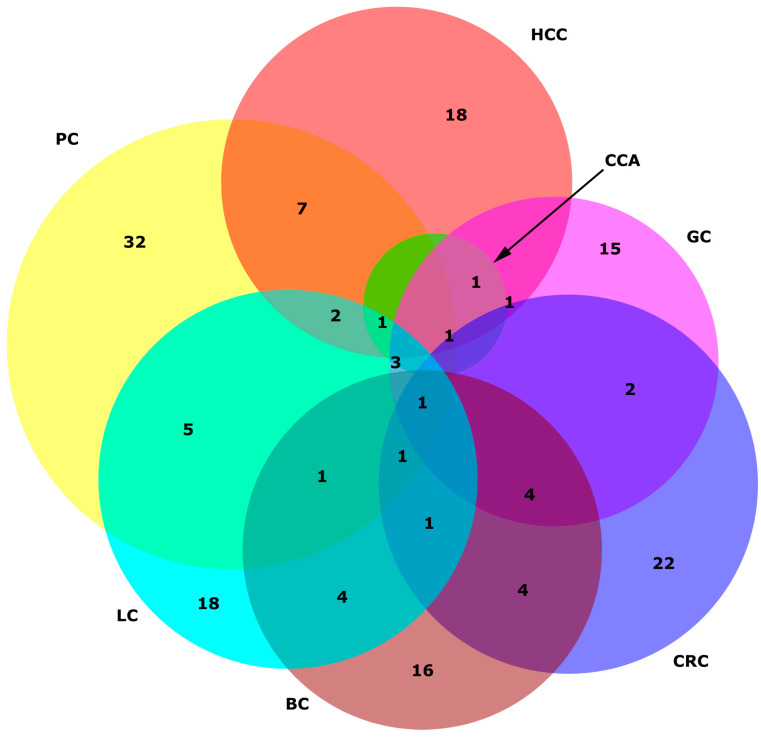
Venn diagram of miRNAs included in miRNA panels presented in this review. Red circle HCC: hepatocellular carcinoma: lime circle CCA: cholangiocarcinoma: blue circle CRC: colorectal cancer: yellow circle PC: pancreatic cancer: fuchsia circle GC: gastric cancer: aqua circle LC: lung cancer: maroon circle BC: breast cancer; ∩: intersection. The intersects among these seven sets are: HCC ∩ CCA ∩ CRC ∩ PC ∩ GC: miR-106b; HCC ∩ CCA ∩ CRC ∩ PC ∩ BC: miR-27a; HCC ∩ CCA ∩ PC ∩ LC: miR-26b; HCC ∩ CCA ∩ GC: miR-10b; HCC ∩ CRC ∩ PC ∩ GC ∩ LC: miR-126; HCC ∩ CRC ∩ PC ∩ GC ∩ LC ∩ BC: miR-21; HCC ∩ CRC ∩ GC: miR-181a; HCC ∩ CRC ∩ LC: miR-375; HCC ∩ PC: miR-30c, miR-222, miR-423, miR-27b, miR-192, miR-885, miR-26a; HCC ∩ PC ∩ GC: miR-221, miR-122; HCC ∩ PC ∩ LC: miR-193b, miR-223; HCC ∩ PC ∩ BC: miR-125b; HCC ∩ LC: miR-214, miR-141, let-7b; HCC ∩ BC: miR-801; CRC ∩ PC: miR-18a, miR-1260b; CRC ∩ PC ∩ GC: miR-20a; CRC ∩ PC ∩ GC ∩ LC ∩ BC: miR-142; CRC ∩ PC ∩ LC ∩ BC: miR-145; CRC ∩ PC ∩ BC: miR-130a; CRC ∩ GC: miR-93, miR-103a; CRC ∩ GC ∩ BC: miR-106a, miR-92a, miR-376c, miR-425; CRC ∩ LC: miR-17, miR-210, miR-23a, miR-19a; CRC ∩ LC ∩ BC: miR-146a; CRC ∩ BC: miR-20b, miR-139, miR-133a, miR-148a; PC ∩ GC ∩ LC: miR-25, miR-29c, miR-340; PC ∩ GC ∩ BC: miR-16; PC ∩ LC: miR-451a, miR-1246, miR-200b, miR-150, miR-125a; PC ∩ LC ∩ BC: miR-574; PC ∩ BC: miR-24, miR-429, miR-92a-2, let-7b; GC ∩ LC: miR-486; GC ∩ LC ∩ BC: miR-19b; GC ∩ BC: miR-451; LC ∩ BC: miR-409, miR-9, miR-30b, let-7a. Created using DeepVenn [104].

**Figure 3:**
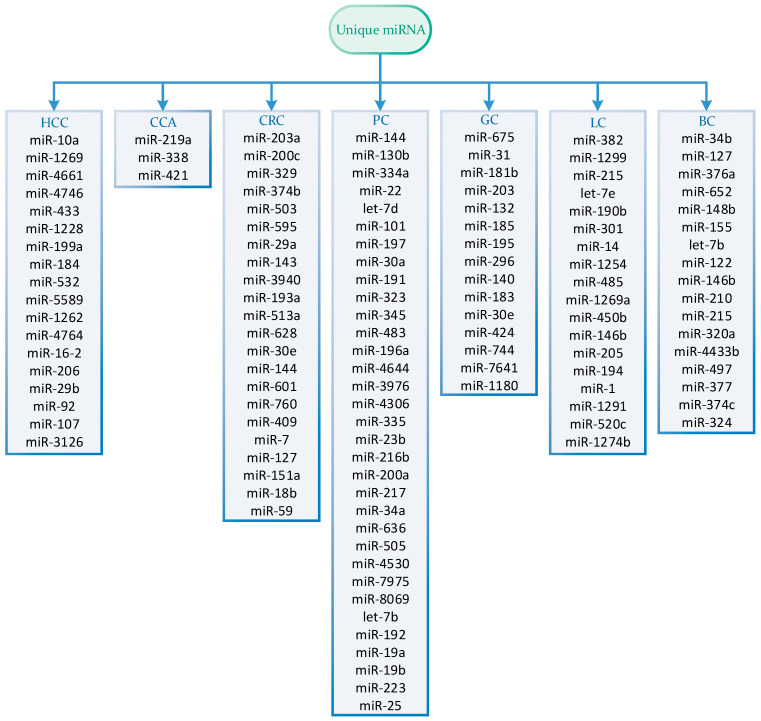
Unique miRNAs presented in each cancer included in miRNA panels from studies selected in this review. HCC: hepatocellular carcinoma; CCA: cholangiocarcinoma; CRC: colorectal cancer; PC: pancreatic cancer; GC: gastric cancer; LC: lung cancer; BC: breast cancer.

From the numerous panels featured in this review, our objective was to identify miRNAs that exhibit specificity for each distinct cancer type, as well as those that are recurrently included in panels across different cancer types. Figure 2 illustrates the intersections among all miRNAs found within panels of various cancer types. It is evident from the figure that certain miRNAs appear to be specific to each cancer type. However, we must acknowledge that the data utilized to generate this figure are sourced from the panels presented within this study. As a result, there remains a possibility that miRNAs designated as unique here may still exhibit differential expression in other cancer types. The cancer-specific miRNAs are presented in Figure 3.

This intriguing revelation underscores the versatility and potential cross-application of certain miRNAs as valuable diagnostic biomarkers across diverse cancer types. The miRNAs highlighted in Figure 2 appear to transcend tissue-specific boundaries, suggesting broader implications in the field of cancer detection and diagnostics.

In our assessment of panels with the largest cohorts, we found that for GC detection, the commercially available panel includes four cancer-specific miRNAs (miR-140, miR-183, miR-30e, and miR-424), while six other miRNAs are common to multiple cancer types [67]. This strategy of selecting miRNAs for panels appears effective, as it takes into account both shared miRNAs across cancer types and those specific to certain cancers. In panels for BC detection, we also identified cancer-specific miRNAs. In the study with the largest cohort, the eight-miRNA panel included four cancer-specific miRNAs (miR-497, miR-377, miR-374c, and miR-324) [93]. In the study with the second largest cohort, four out of five miRNAs were cancer-specific (miR-122, miR-146b, miR-210, and miR-215) [94].

The PC panel included three cancer-specific miRNAs (miR-34a, miR-636, and miR-505) out of ten miRNAs in the panel [55]. Similarly, the CCA panel contained three out of seven miRNAs that were cancer-specific (miR-219a, miR-338, and miR-421) [35]. In LC, the first three largest cohort studies each included at least one cancer-specific miRNA: miR-1 in a five-miRNA panel in the largest cohort study [77], miR-190b in a six-miRNA panel in the second-largest cohort study [78], and miR-194 in a three-miRNA panel in the third study [79]. Interestingly, we did not observe cancer-specific miRNAs in HCC [18] and CRC [39], as the most statistically reliable panels did not include the miRNAs we identified as cancer-specific.

This observation opens up exciting avenues for further research and exploration into commonalities and shared molecular signatures that may underlie various cancer types, ultimately paving the way for more universal and robust cancer detection strategies.

## 7. Conclusions and Future Perspectives

We performed a systematic review of the literature of miRNA panels able to detect primary liver cancers, HCC and CCA, and liver-metastasizing cancer, which includes CRC, GC, PC, LC and BC. The ability to distinguish between primary liver cancers and metastatic liver cancers presents an intricate diagnostic challenge of paramount importance. Our research approach was centered on providing a comprehensive overview of existing studies and their findings. However, we recognize that further in-depth research is essential to unravel the intricacies of miRNA deregulation in specific cancer types as compared to others. Specifically, the identification of miRNAs that exhibit distinct deregulation patterns in specific cancer types compared to their counterparts holds immense potential. These identified miRNAs will be fundamental for the development of miRNA panels tailored for discriminating between different cancer types. This approach assumes a critical role in cancer diagnostics, particularly in the context of distinguishing between primary liver cancers and liver-metastasizing cancers. The ability to make this distinction is pivotal, as it has direct implications for clinical management and prognosis, particularly in cases where the origin of the cancer is initially uncertain.

In essence, our systematic review of the literature serves as a foundational step, highlighting the need for further research endeavors that focus on pinpointing specific miRNAs linked to distinct cancer types. With these insights, we can develop miRNA panels that hold the promise of significantly enhancing our ability to differentiate between primary and metastatic liver cancers, ultimately leading to more accurate diagnoses and tailored treatment strategies. Although most of studies have been made on primary tumors and healthy tissue, possible miRNA panels that hold the promise of significantly enhancing our ability to differentiate between primary and metastatic liver cancers could be proposed, ultimately leading to more accurate diagnoses and personalized treatment strategies.

## Figures and Tables

**Table 1:** An overview of circulating miRNA panels for detection of HCC.

miRNA Panel	Sample Type	Number of Samples	Expression	Statistics	Reference
miRNA Panels					
miR-122	Plasma	457 HCC, 167 HC	↑	AUC = 0.941	[18]
miR-192			↑		
miR-21			↑		
miR-223			↓		
miR-26a			↓		
miR-27a			↓		
miR-801			↓		
miR-206	Serum	261 HCC, 173 HC	↑	AUC = 0.887 (95% CI = 0.850–0.918) Sensitivity = 85.55% Specificity = 73.3%	[19]
miR-141-3p			↑		
miR-433-3p			↑		
miR-1228-5p			↑		
miR-199a-5p			↓		
miR-122-5p			↓		
miR-192-5p			↓		
miR-26a-5p			↓		
miR-214-5p	Serum	224 HCC, 84 HC	↓	AUC = 0.95 with 95% CI Sensitivity = 83.2% Specificity = 96.9% Accuracy = 86.8%	[20]
miR-125b			↓		
miR-1269			↑		
miR-375			↓		
miR-27b-3p	Serum	212 HCC, 110 HC	↑	AUC = 0.823 (*p* < 0.0001)	[21]
miR-192-5p			↑		
miR-375	Serum	149 HCC, 149 HC	↑	AUC = 0.995 (95% CI: 0.985–1)	[22]
miR-10a			↑		
miR-122			↑		
miR-423			↑		
miR-4661-5p	Serum exosomes	84 HCC, 26 HC	↑	AUC = 0.942 (95% CI = 0.895–0.972) Sensitivity = 84.5% Specificity = 89.3% PPV = 88.8% NPV = 85.2%	[23]
miR-4746-5p			↑		
miR-126	Serum	34 HCC, 25 HC	↑	AUC = 1.00 SE = 0 *p*-value < 0.001	[24]
miR-21			↑		
miR-30c			↑		
miR-193b			↑		
miR-122			↑		
miR-222			↑		
miR-125b			↑		
miR-10b	Serum	27 HCC, 50 HC	↑	AUC = 0.94 (95% CI: 0.89–0.99)	[25]
miR-181a			↓		
miR-106b			↑		
miRNA + lncRNA + mRNA panels					
miR-16-2	Serum	78 HCC, 42 HC	↑	Sensitivity = 79.5% Specificity = 100%	[26]
miR-21-5p			↑		
lncRNA-CTBP			↑		
mRNA LAMP2			↑		
miR-1262	Serum exosomes	60 HCC, 18 HC	↓	Sensitivity = 100% Specificity = 76.7% PPV = 81.1% NPV = 100% Accuracy = 88.3%	[27]
lncRNA-RP11-513I15.6			↓		
mRNA RAB11A			↓		
miR-4764-5p	Serum	49 HCC, 36 HC	↓	Sensitivity = 100% Specificity = 76.7% PPV = 81.1% NPV = 100% Accuracy = 88.3%	[28]
lncRNA-RP11-156p1.3			↓		
mRNA RFTN1			↓		
miRNA + AFP panels					
miR-122	Serum	192 HCC, 95 HC	↑	AUC = 1	[29]
miR-885-5p			↑		
miR-29b			↓		
AFP					
miR-92-3p	Serum	115 HCC, 40 HC	↑	AUC = 0.988	[30]
miR-107			↑		
miR-3126-5p			↓		
AFP					
miR-125b	Serum	90 HCC, 30 HC	↓	AUC = 0.936 (CI = 0.878ߝ0.995) Sensitivity = 0.907 Specificity = 0.933	[31]
miR-223			↓		
miR-27a			↓		
miR-26a			↓		
AFP					
miR-206	Plasma	38 HCC, 20 HC	↑	AUC = 0.989 (CI = 0.919-1.000)	[32]
miR-126			↓		
AFP					

HCC: hepatocellular carcinoma; HC: healthy controls; AFP: α-fetoprotein; AUC: area under receiver operating characteristic curve; SE: standard deviation; PPV: positive predictive value; NPV: negative predictive value; CI: confidence interval; ↑: upregulated expression; ↓: down-regulated expression.

**Table 2:** An overview of circulating miRNA panels for detection of CCA.

miRNA Panel	Sample Type	Number of Samples	Expression	Statistics	Reference
miR-10b-3p	plasma	48 CCA, 20 HC	↑	AUC = 0.781 (95% CI: 0.585–0.914) Sensitivity = 83.3% Specificity = 75.0% PPV = 71.4% NPV = 85.7%	[35]
miR-26b-3p			↑		
miR-27a-3p			↑		
miR-106b-3p			↑		
miR-219a-3p			↑		
miR-338-5p			↑		
miR-421			↑		

CCA: cholangiocarcinoma; HC: healthy controls; AUC: area under receiver operating characteristic curve; PPV: positive predictive value; NPV: negative predictive value; CI: confidence interval; ↑: upregulated expression; ↓: down-regulated expression.

**Table 3:** An overview of circulating miRNA panels for detection of CRC.

miRNA Panel	Sample Type	Number of Samples	Expression	Statistics	Reference
miRNA panels					
miR-23a-3p	Serum	427 CRC, 276 HC	↑	AUC = 0.877 Sensitivity = 81.4% Specificity = 81% PPV = 80.6% NPV = 81.8% Accuracy = 81.2%	[39]
miR-27a-3p			↑		
miR-142-5p			↑		
miR-376c-3p			↑		
miR-19a-3p	Serum	196 CRC, 138 HC	↑	AUC = 0.87	[40]
miR-21-5p			↑		
miR-425-5p			↑		
miR-145	Serum	175 CRC, 130 HC	↓	AUC = 0.886 (95% CI = 0.850–0.921)	[41]
miR-106a			↑		
miR-17-3p			↑		
miR-27a	Exosomes	170 CRC, 130 HC	↑	AUC = 0.801	[42]
miR-130a			↑		
miR-193a-5p	Plasma	149 CRC, 110 HC	↑	AUC = 0.88 (95% CI = 0.82–0.93)	[43]
miR-210			↑		
miR-513a-5p			↑		
miR-628-3p			↑		
miR-103a-3p	Plasma	139 CRC, 132 HC	↑	AUC = 0.895	[44]
miR-127-3p			↑		
miR-151a-5p			↑		
miR-17-5p			↑		
miR-181a-5p			↑		
miR-18b-5p			↑		
miR-30e-3p	Serum	137 CRC, 145 HC	↑	AUC = 0.883 Sensitivity = 0.800 Specificity = 0.787	[45]
miR-146a-5p			↑		
miR-148a-3p			↓		
miR-203a-3p	Serum	135 CRC, 135 HC	↑	AUC = 0.893 Sensitivity = 81.25% Specificity = 73.33%	[46]
miR-145-5p			↓		
miR-375-3p			↓		
miR-200c-3p			↓		
miR-18a	Plasma	130 CRC, 244 HC	↑	AUC = 0.745 (95% CI = 0.708–0.846)	[47]
miR-20a			↑		
miR-21			↑		
miR-29a			↑		
miR-92a			↑		
miR-106b			↑		
miR-133a			↑		
miR-143			↑		
miR-145			↑		
miR-409-3p	Plasma	124 CRC, 117 HC	↑	AUC = 0.897	[48]
miR-7			↓		
miR-93			↓		
miR-144-3p	Plasma	101 CRC, 134 HC	↓	Sensitivity = 93.8% Specificity = 91.3%	[49]
miR-425-5p			↓		
miR-1260b			↓		
miR-601	Plasma	90 CRC, 58 HC	↓	AUC = 0.792 Sensitivity = 83.3% Specificity = 69.1%	[50]
miR-760			↓		
miR-126	Plasma	50 CRC, 150 HC	↓	AUC = 0.906	[51]
miR-139			↓		
miR-143			↓		
miR-595			↑		
miRNA + lncRNA + mRNA panels					
miR-20b-5p	Plasma	597 CRC, 585 HC	↑	AUC = 0.954 (95% CI = 0.913–0.994)	[52]
miR-329-3p			↑		
miR-374b-5p			↑		
miR-503-5p			↑		
lncRNA-XLOC_001120			↑		
lncRNA-ENSG00000243766.2			↑		
miR-3940-5p	Plasma	70 CRC *	↓	Sensitivity = 100% Specificity = 88.6% PPV = 100% NPV = 85% Accuracy = 93.07%	[53]
lncRNA-SNHG14			↑		
mRNA-NAP1L2			↑		
miR-59	Serum	70 CRC, 20 HC	↓	Sensitivity = 100% Specificity = 61.7% PPV = 75.3% NPV = 100% Accuracy = 83.1%	[54]
lncRNA-RP11-909B2.1			↑		
mRNA L3MBTL1			↑		

CRC: colorectal carcinoma; HC: healthy controls; AUC: area under receiver operating characteristic curve; PPV: positive predictive value; NPV: negative predictive value; CI: confidence interval; ↑: upregulated expression; ↓: down-regulated expression; *: it was impossible to deduce the number of healthy controls included in the study.

**Table 4:** An overview of circulating miRNA panels for detection of PC.

miRNA Panel	Sample Type	Number of Samples	Expression	Statistics	Reference
miRNA panels					
miR-122	Plasma	409 PC, 312 HC	↑	AUC = 0.93 (95% CI = 0.90–0.96)	[55]
miR-34a			↑		
miR-145			↑		
miR-636			↑		
miR-223			↑		
miR-26b			↑		
miR-885-5p			↑		
miR-150			↑		
miR-126			↑		
miR-505			↑		
miR-122-5p	Plasma	216 PC, 220 HC	↑	AUC = 0.937	[56]
miR-125b-5p			↑		
miR-192-5p			↑		
miR-193b-3p			↑		
miR-221-3p			↑		
miR-27b-3p			↑		
miR-30c-5p	Plasma	168 PC, 124 HC	↑	AUC = 0.93	[57]
miR-340-5p			↑		
miR-335-5p			↑		
miR-23b-3p			↑		
miR-142-3p			↑		
miR-145-5p			↑		
miR-200b-3p			↑		
miR-429			↑		
miR-1260b			↑		
miR-145-3p			↑		
miR-216b-5p			↑		
miR-200a-3p			↑		
miR-217-5p			↑		
let-7b-5p	Plasma	129 PC, 107 HC	↑	AUC = 0.910	[58]
miR-192-5p			↑		
miR-19a-3p			↑		
miR-19b-3p			↑		
miR-223-3p			↑		
miR-25-3p			↑		
miR-574-3p	Plasma	90 PC, 154 HC	↑	AUC = 0.96 (95% CI = 0.92–1.00)	[59]
miR-885-5p			↑		
miR-144-3p			↓		
miR-130b-3p			↑		
miR-334a-5p			↑		
miR-24-3p			↑		
miR-106b-5p			↓		
miR-22-5p			↑		
miR-451a			↓		
let-7d-3p			↑		
miR-101-3p			↓		
miR-26a-5p			↓		
miR-197-3p			↑		
miR-423-3p			↑		
miR-122-5p			↑		
miR-125a-3p	Plasma	77 PC, 65 HC	↑	AUC = 0.862	[60]
miR-4530			↑		
miR-92a-2-5p			↑		
miRNA + CA19-9 panels					
miR-16	Serum	471 PC, 248 HC	*	AUC = 0.94 (95% CI = 0.90–0.97) Sensitivity = 85 %Specificity = 98% Accuracy = 89%	[61]
miR-18a					
miR-20a					
miR-24					
miR-25					
miR-27a					
miR-29c					
miR-30a-5p					
miR-191					
miR-323-3p					
miR-345					
miR-483-5p					
CA19-9					
miR-16	Plasma	138 PC, 68 HC	↑	AUC = 0.979 (95% CI = 0.962–0.996)	[62]
miR-196a			↑		
CA19-9			↑		
miR-34a-5p	Plasma	136 PC, 73 HC	↑	AUC = 0.94 (95% CI = 0.89–0.98)	[63]
miR-130a-3p			↑		
miR-222-3p			↑		
CA19-9			↑		
miR-130a-3p	Plasma	68 PC, 61 HC	↑	AUC = 0.986 (95% CI = 0.972–1.000)	[64]
miR-21-5p			↑		
miR-223-3p			↑		
miR-7975			↑		
miR-8069			↑		
CA19-9			↑		
miRNA + protein panels					
miR-1246	Serum	131 PC, 20 HC	↑	Sensitivity = 100% (95% CI = 95%–100%) Specificity = 80% (95% CI: 67%–90%)	[65]
miR-4644			↑		
miR-3976			↑		
miR-4306			↑		
CD44v6			↑		
Tspan8			↑		
MET			↑		
CD104			↑		

PC: pancreatic cancer; HC: healthy controls; CA19-9: carbohydrate antigen 19-9; AUC: area under receiver operating characteristic curve; CI: confidence interval; ↑: upregulated expression; ↓: down-regulated expression; *: the data on down- or upregulation of miRNA was not presented in publication.

**Table 5:** An overview of circulating miRNA panels for detection of GC.

miRNA Panel	Sample Type	Number of Samples	Expression	Statistics	Reference
miRNA panels					
miR-140	Serum	424 GC, 468 HC	*	AUC = 0.92 (95% CI = 0.88–0.96) Sensitivity = 87.0% (95% CI = 0.794–0.925) Specificity = 68.5% (95% CI = 0.670–0.698)	[67]
miR-183					
miR-30e					
miR-103a					
miR-126					
miR-93					
miR-142					
miR-21					
miR-29c					
miR-424					
miR-181a					
miR-340					
miR-10b-5p	Serum/exosomes	205 GC, 167 HC/30 GC, 28 HC	↑	AUC = 0.702	[68]
miR-132-3p			↑		
miR-185-5p			↑		
miR-195-5p			↑		
miR-20a-3p			↑		
miR-296-5p			↑		
miR-19b-3p	Serum exosomes	130 GC, 130 HC	↑	AUC = 0.814	[69]
miR-106a-5p			↑		
miR-16	Plasma	124 GC, 160 HC	↑	AUC = 0.812	[70]
miR-25			↑		
miR-92a			↑		
miR-451			↑		
miR-486-5p			↑		
miR-21	Plasma	115 GC, 60 HC	↑	AUC = 0.887 (95% CI = 0.83–0.943) Sensitivity = 84.8% Specificity = 79.2%	[71]
miR-93			↑		
miR-106a			↑		
miR-106b			↑		
miR-21	Serum	92 GC, 89 HC	↑	AUC = 0.919 (95% CI = 0.863-0.975)	[72]
miR-31			↓		
miR-92a			↓		
miR-181b			↓		
miR-203			↓		
miR-221	Serum	82 GC, 82 HC	↑	Sensitivity = 82.4% Specificity = 58.8%	[73]
miR-744			↑		
miR-376c			↑		
miR-7641	Plasma	62 GC, 90 HC	↓	AUC = 0.799 (95% CI = 0.691–0.908) *p* < 0.001	[74]
miR-425-5p			↓		
miR-1180-3p			↓		
miR-122-5p			↓		
miRNA + lncRNA panels					
miR-675-5p	Plasma	62 GC, 40 HC	↓	AUC = 0.927 (95% CI = 0.85–0.96) *p* < 0.0001 Sensitivity = 88.78% Specificity = 85%	[75]
H19			↑		
MEG3			↓		

GC: gastric cancer; HC: healthy controls; AUC: area under receiver operating characteristic curve; CI: confidence interval; ↑: upregulated expression; ↓: down-regulated expression; *: the data on down- or upregulation of miRNA was not presented in publication.

**Table 6:** An overview of circulating miRNA panels for detection of LC.

miRNA Panel	Sample Type	Number of Samples	Expression	Statistics	Reference
miRNA panels					
let-7a-5p	Serum	744 NSCLC, 944 HC	↓	AUC = 0.973 (95% CI = 0.947–0.987)	[77]
miR-375			↓		
miR-1-3p			↑		
miR-1291			↑		
miR-214-3p			↑		
miR-17	Plasma	676 NSCLC, 456 HC	↓	AUC = 0.873 (95% CI = 0.843–0.899) Sensitivity = 81% Specificity = 80%	[78]
miR-190b			↓		
miR-19a			↓		
miR-19b			↓		
miR-26b			↓		
miR-375			↑		
miR-451a	Serum exosomes	434 LUAD, 149 HC	↑	AUC = 0.965	[79]
miR-194-5p			↑		
miR-486-5p			↑		
miR-193b	Serum	154 NSCLC, 45 HC	↑	AUC = 0.993 (95% CI 0.979–1.000) *p* < 0.001	[80]
miR-301			↑		
miR-14			↑		
miR-200b			↑		
miR-9-3p	Serum exosomes	147 NSCLC, 149 HC	↑	AUC = 0.878	[81]
miR-205-5p			↑		
miR-210-5p			↑		
miR-1269a			↑		
miR-146b	Serum	128 NSCLC, 30 HC	↑	AUC = 0.96 Accuracy = 92.005	[82]
miR-205			↑		
miR-29c			↑		
miR-30b			↑		
miR-340	Plasma	120 NSCLC, 120 HC	↓	AUC = 0.862 Sensitivity = 78.33% Specificity = 77.5%	[83]
miR-450b-5p			↑		
miR-125a-5p	Serum	118 NSCLC, 135 HC	↓	AUC = 0.936 Sensitivity = 87.5% Specificity = 87.5%	[84]
miR-25			↓		
miR-126			↓		
miR-142-5p	Serum	112 LUAD, 120 HC	↑	AUC = 0.933 (95% CI = 0.884–0.965) Sensitivity = 82.93% Specificity = 96.67%	[85]
miR-409-3p			↓		
miR-223-3p			↑		
miR-146a-5p			↓		
let-7b-5p	Plasma	46 NSCLC, 41 HC	↓	AUC = 0.868 Sensitivity = 80% Specificity = 80%	[86]
let-7e-5p			↑		
miR-23a-3p			↓		
miR-486-5p			↓		
miR-215-5p	Serum	39 NSCLC, 32 HC	↓	AUC = 0.8013 Sensitivity = 67% Specificity = 68%	[87]
miR-1299			↓		
miR-205-5p			↑		
miR-1246			↑		
miR-520c-3p	Serum exosomes	36 NSCLC, 36 HC	↑	AUC = 0.857 (95% CI, 0813–0.901) *p* < 0.0001	[88]
miR-1274b			↑		
miR-145	Serum	30 NSCLC, 20 HC	↑	AUC = 1 Sensitivity = 100% Specificity 100%	[89]
miR-382			↑		
miR-21	Serum	28 NSCLC, 17 HC	↑	AUC = 0.91 (95% CI = 0.80–1.0)	[90]
miR-223			↓		
miR-205-5p	Serum	20 SCLC, 32 HC	↓	AUC = 0.948 Sensitivity = 90.00% Specificity = 93.75%	[87]
miR-1299			↓		
miR-215-5p			↓		
miR-141-3p			↓		
miR-200b-5p			↓		
miRNA + lncRNA panels					
miR-1254	Serum	156 NSCLC, 107 HC	↓	AUC = 0.844 (95% CI = 0.778–0.91) Sensitivity = 93.3% Specificity = 73.2%	[91]
miR-485-5p			↓		
miR-574-5p			↓		
MALAT1			↓		
miR-150	Serum	30 NSCLC, 15 HC	↓	AUC = 0.784 Sensitivity = 80% Specificity = 80%	[92]
linc00673			↑		

LC: lung cancer; NSCLC: non-small cell lung cancer cancer; SCLC: small cell lung cancer; LUAD: lung adenocarcinoma; HC: healthy controls; AUC: area under receiver operating characteristic curve; CI: confidence interval; ↑: upregulated expression; ↓: down-regulated expression.

**Table 7:** An overview of circulating miRNA panels for detection of BC.

miRNA Panel	Sample Type	Number of Samples	Expression	Statistics	Reference
miRNA panels					
miR-133a-3p	Serum	540 BC, 502 HC	↑	AUC = 0.915 Accuracy = 82.3% Sensitivity = 72.2% Specificity = 91.5%	[93]
miR-497-5p			↑		
miR-24-3p			↑		
miR-125b-5p			↑		
miR-377-3p			↓		
miR-374c-5p			↓		
miR-324-5p			↓		
miR-19b-3p			↓		
let-7b-5p	Plasma	257 BC, 257 HC	↑	AUC = 0.978	[94]
miR-122-5p			↑		
miR-146b-5p			↑		
miR-210-3p			↑		
miR-215-5p			↑		
miR-127-3p	Plasma	247 BC, 140 HC	↑	AUC = 0.81 (95% CI = 0.75–0.88)	[95]
miR-376a			↑		
miR-652			↑		
miR-148b			↑		
miR-376c			↑		
miR-409-3p			↑		
miR-801			↑		
miR-106a-5p	Serum	204 BC, 202 HC	↑	AUC = 0.93 (95% CI = 0.911–0.964) Sensitivity = 87% Specificity = 89%	[96]
miR-19b-3p			↑		
miR-20b-5p			↑		
miR-92a-3p			↑		
miR-106a-3p	Plasma	200 BC, 200 HC	↑	AUC = 0.88 (95% CI = 0.855–0.923) Sensitivity = 82% Specificity = 79%	[96]
miR-106a-5p			↑		
miR-20b-5p			↑		
miR-92a-2-5p			↑		
miR-92a	Serum	164 BC, 132 HC	↑	AUC = 0.91	[97]
miR-133a			↑		
miR-9-5p	Serum	135 BC, 125 HC	↑	AUC = 0.880 Sensitivity = 86.25% Specificity = 81.25%	[98]
miR-34b-3p			↓		
miR-146a-5p			↓		
miR-9	Plasma	62 BC, 20 HC	↑	AUC = 0.88 (95% CI = 0.78–0.99) Sensitivity = 96.8% Specificity = 80%	[99]
miR-16			↑		
miR-21			↑		
miR-429			↑		
miR-451	Serum	60 BC, 29 HC	↓	AUC = 0.953 Sensitivity = 94.7% Specificity = 82.8%	[100]
miR-148a			↓		
miR-27a			↓		
miR-30b			↓		
miR-145	Plasma	41 BC, 32 HC	↑	AUC = 0.97 (95% CI = 0.929–1.000) Sensitivity 97% Specificity 91%	[101]
miR-425-5p			↑		
miR-139-5p			↑		
miR-130a			↑		
miR-142-5p	Serum	31 BC, 16 HC	↑	AUC = 0.8387 Sensitivity = 93.33% Specificity = 68.75%	[102]
miR-320a			↑		
miR-4433b-5p			↑		
miRNA + lncRNA panels					
let-7a	Serum	158 BC, 107 HC	↓	AUC = 0.968 PPV = 0.97 NPV = 0.85	[103]
miR-155			↑		
miR-574-5p			↑		
MALAT1			↑		

BC: breast cancer; HC: healthy controls; AUC: area under receiver operating characteristic curve; CI: confidence interval; ↑: upregulated expression; ↓: down-regulated expression.

## Data Availability

Not applicable.
